# Establishment of a risk prediction model for hydrocephalus complicated by neonatal bacterial meningitis

**DOI:** 10.1186/s12879-025-11517-x

**Published:** 2025-09-26

**Authors:** Yue Tianrui, Bao Lingyun, Gao Jin

**Affiliations:** Kunming Children’s Hospital, Kunming, 650000 China

**Keywords:** Neonatal bacterial meningitis, Hydrocephalus, Complications, Risk factors, Nomogram

## Abstract

**Background:**

Hydrocephalus is a severe complication of neonatal bacterial meningitis (NBM), threatening the health and quality of life of neonates, affecting the outcome of nervous system development, and leading to neurological sequelae, such as movement disorders, hearing impairment, mental retardation, and epilepsy. Improvement in prognosis is closely related to early identification and active treatment.

**Objective:**

To find the independent risk factors of NBM complicated with hydrocephalus, to construct the related risk prediction model and validate it, in order to provide help for clinicians to identify the children with high risk of hydrocephalus at an early stage, to guide clinical decision-making and improve prognosis.

**Methods:**

528 children with NBM hospitalized in Kunming Children’s Hospital from January 2019 to December 2022 were selected. After excluding 46 patients with incomplete medical records And 1 death case, 481 patients remained. They were randomly divided into a training set (*n* = 337) and a validation set (*n* = 144) (the division ratio was 7:3) by using the split function in R language. The basic information, cerebrospinal fluid routine biochemistry, blood routine, blood culture, imaging findings, and other indicators of the children were collected. Determination of whether hydrocephalus was complicated based on the child’s brain magnetic resonance imaging or CT. LASSO regression was used to screen independent risk factors for NBM complicated by hydrocephalus, And independent risk factors were Analyzed by using multivariate logistic regression. The risk prediction model for NBM complicated by hydrocephalus was constructed by using the analysis results, and a nomogram was created. The model was internally validated based on the cases in the training and internal validation sets. A total of 132 children with NBM who were hospitalized at Peking University First Hospital from January 2006 to December 2021 were included in the study. After excluding 2 cases with incomplete medical records, the remaining 130 cases were used as external validation cases to externally validate the model.

**Results:**

Twenty predictive variables were screened out by LASSO regression analysis, including NBM type, BW, age of onset, pregnancy complications, gestational age, birth asphyxia, umbilical cord, amniotic fluid, maximum body temperature, vomiting, convulsions, anterior fontanel, blood culture, PLT, peak value of WBC, peak value of N, peak value of PLT, CSF multinucleated percentage peak, lowest value of CSF glucose, and intracranial hemorrhage. The results of multifactorial Logistic regression analysis after oversampling showed that the significant risk factors were intracranial hemorrhage (OR = 6.922, *P* < 0.001), anterior fontanel (OR = 8.002, *P* < 0.001), lowest value of CSF glucose (OR = 0.416, *P* < 0.001), gestational week (OR = 0.870, *P* = 0.0088), maternal pregnancy complications (OR = 0.284, *P* = 0.0118), convulsions (OR = 2.906, *P* = 0.0178), amniotic fluid (OR = 2.417, *P* = 0.0263), and CSF multinucleated percentage peak (OR = 1.011, *P* = 0.0350). There was no statistically significant difference between convulsions, maternal pregnancy complications and CSF multinucleated percentage peak in binary logistic regression. Therefore, a nomogram risk prediction model was created with the remaining five predictive variables. The area under the ROC curve (AUC) of the training set after weighting was 0.925 (95%CI = 0.899–0.951), the internal validation set was 0.894 (95%CI = 0.829–0.959), And the external validation set was 0.758 (95%CI = 0.677–0.839); the goodness-of-fit test showed that the training set *P* = 0.431, internal validation set *P* = 0.224, and external validation set *P* = 0.176. Decision curve analysis (DCA) showed that the net benefit of the model was higher than the net benefit at the extremes in a large range of thresholds in the training set, internal validation set, and external validation set.

**Conclusion:**

The Nomogram risk prediction model established in this study, which includes five indicators of the lowest CSF glucose level, combined intracranial hemorrhage, anterior fontanel, gestational week, and amniotic fluid, can early predict the risk of NBM complicating hydrocephalus.

The immune system and blood-brain barrier of neonates are not yet fully developed, which increase their vulnerability to pathogen invasion. The incidence of NBM is the highest among all age groups [1–4]. Moreover, NBM is a severe intracranial infectious disease in the neonatal population, with a high incidence And mortality rate globally. Currently, with the continuous progress of medical technology, the incidence rate in developed countries has dropped to 0.3 cases per 1000 Live births, And the mortality rate has dropped to 10% - 15% [5]. In developing countries, the incidence rate has dropped to 0.8 - 6.1 cases per 1000 Live births, And the mortality rate has dropped to 40% - 60%. However, the incidence of hydrocephalus, a serious complication, has not shown a significant downward trend [6–10].

Hydrocephalus is a severe complication of NBM, with a prevalence rate of 9%−35%. In the course of infection, purulent exudate adheres to the surface of the arachnoid or ventricular meninges, preventing the circulation of cerebrospinal fluid (CSF) or decreasing the ability of the arachnoid granules to absorb CSF, which can eventually lead to hydrocephalus. [11–14]. If NBM complicated by hydrocephalus is not promptly diagnosed and aggressively treated, it may cause irreversible damage to the neonatal brain and neurological sequelae, leading to a range of cognitive and behavioral disorders.

Nomogram is a visual risk regression model that has emerged as an effective tool for predicting patient prognosis or clinical events [15–18]. At present, there are not many domestic and international studies on the prediction model of the risk of hydrocephalus complicating NBM, so establishing a prediction model that can be conveniently used in the clinic to provide clinicians with a tool for early prediction of the occurrence of hydrocephalus will be conducive to the timely adoption of interventions to improve the prognosis of the children.

## Study design

### Research objectives

This retrospective study used data from the clinical records of children diagnosed with NBM at the Kunming Children’s Hospital And Peking University First Hospital. A total of 528 children with NBM who were hospitalized at Kunming Children’s Hospital from January 2019 to December 2022 were enrolled. After excluding 46 patients with incomplete medical records And 1 death case, 481 patients remained. Another 132 children with NBM hospitalized in Peking University Hospital from January 2006 to December 2021 were collected. After excluding 2 patients with incomplete medical records, 130 patients remained.

### Inclusion and exclusion criteria

All data were obtained from electronic historical medical records.

Inclusion criteria: Children diagnosed with NBM and presenting with manifestations or examination results related to hydrocephalus during NBM (generally within 1–2 weeks after the onset).

Diagnostic criteria for NBM: According to the diagnostic criteria in the fifth edition of Practical Neonatology [19]. (1) Age: ≤ 28 days; (2) Positive cerebrospinal fluid bacterial culture or smear Gram staining; (3) Changes in cerebrospinal fluid (CSF) routine and biochemical examinations: ① Protein: >1.7 g/L; ② White blood cell (WBC) count: ≥ 20 × 10⁹/L; ③ Multinucleated percentage > 57% − 61%; ④ Glucose: < 2.2mmol/L or lower than 40% of the current blood glucose; (4) Clinical symptoms and signs of NMB.

Diagnostic criteria for hydrocephalus: Cranial ultrasound, cranial computed tomography (CT) or magnetic resonance imaging (MRI) met one of the following: ① Evans index (maximum width of the frontal horn/maximum biparietal diameter at the same level) >0.3;② Maximum distance between the tips of the two anterior horns of the lateral ventricles >45 mm༛③ The width of the third ventricle is > 6 mm༛④ The width of the third ventricle is > 12 mm.

Inclusion criteria for intracranial hemorrhage [14]: (1) suspected intracranial hemorrhage after cranial ultrasonography screening; (2) papile grade ≥ Ⅱ indicated by cranial magnetic resonance imaging(MRI) or cranial computed tomography(CT); (3) complete clinical pathological and imaging data.

**Fig. 1:**
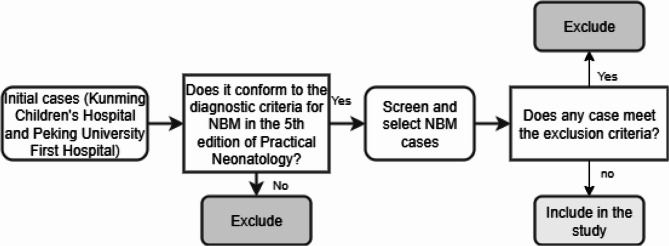
Schematic diagram of inclusion and exclusion process for NBM cases

The exclusion criteria were as follows: (1) various factors leading to incomplete or missing case information (e.g., abandonment of treatment, transfer to other hospitals, etc.); (2) it could not yet be ruled out that there might be concurrent comorbidities with other central nervous system disorders; (3) comorbidities with severe congenital cranio-cerebral malformations (spinal meningeal bulge, neural tube developmental anomalies, etc.), neural tube developmental defects, congenital middle cerebral conduit blockage, head trauma, choroid plexus papillomas, and arachnoid cysts, Dandy-Walker syndrome, Jobert syndrome, etc. (Fig 1).

### Data integration

The data collected from children in this study for modeling mainly included basic information, clinical manifestations, and laboratory results. The basic information mainly includes gender, NBM type (early-onset or late-onset), age of onset, mother’s age, gestational age, birth asphyxia, umbilical cord, amniotic fluid, pregnancy complications. The clinical manifestations mainly include the maximum body temperature, body temperature recurrence > 3 days, vomiting, poor appetite, difficulty or shortness of breath, convulsions, anterior fontanel, muscle tension. The laboratory results mainly include blood culture, multiple drug resistance in blood culture, platelet (PLT), the peak value of blood WBC, the peak value of blood neutrophils (N), peak value of C-reactive protein (CRP), CSF culture, multiple drug resistance in CSF culture, cerebrospinal fluid WBC peak, peak percentage of CSF multinucleated cells, peak value of CSF protein, the lowest value of CSF glucose, combined intracranial hemorrhage, neutrophil platelet ratio. The peak value is the highest value of the indicator presented during the disease in the child.

The inclusion criteria for intracranial hemorrhage in data collection were [14] (1) suspected intracranial hemorrhage after cranial ultrasound screening, (2) cranial MRI or cranial CT was performed to suggest Papile grade ≥ II, and (3) complete clinicopathologic and imaging data were available.

After organizing the included data from Kunming Children’s Hospital, they were entered into R 4.4.0 software And randomly divided according to the ratio of 7:3 by applying the split function in the caTools function package (in which the parameter was set to SplitRatio = 0.7), so as to make them belong to the training set (70%) and the internal validation set (30%), respectively. All the included data materials from the Peking University First University were used as an external validation set.

### Statistical methods

The statistical Analysis was performed by using R 4.4.0 software. Measurement data were expressed as mean (standard deviation). The count data were expressed as frequency or percentage. The baseline data of the training and validation sets were compared by using the X^2^ or t-tests. Propensity score weighting was used to balance differences in baseline characteristics of cases from different centers. A 1:1 oversampling was applied to balance the sample imbalance caused by the lower incidence of NBM-complicated hydrocephalus in the training set.The risk factors for NBM-complicated hydrocephalus were screened by LASSO regression in the R language, and then further screened by multifactorial logistic regression analysis, and finally, the predictive model was constructed and plotted as a nomogram (columnar plot). The training set and validation set models were then evaluated by subject work (ROC) curves, goodness-of-fit tests, calibration curves, clinical decision curves (DCA), cross-validation and Bootstrap method. Differences were considered statistically significant at *P* < 0.05.

## Results

### Baseline comparison

A total of 481 children with NBM at Kunming Children’s Hospital were ultimately included in this study and randomly divided into a training set (337 cases) and an internal validation set (144 cases) at a ratio of 7:3. Among them, 60 were complicated by hydrocephalus, accounting for 12.47% of the total number of cases. At the same time, 130 children with NBM from Peking University First Hospital were included as An external validation set, among which 51 cases were complicated by hydrocephalus, accounting for 38.64% of the total number of cases. The three datasets differed statistically on variables such as gestational week, whether the amniotic fluid was fecal-stained at birth, whether there were convulsions during the course of the disease, whether there was a bulging fontanel during the course of the disease, and the CSF glucose nadir and peak CSF protein values (Table 1). These differences indicate that the condition of children admitted to the Peking University First Hospital was more severe and complex compared with that of children admitted to the Kunming Children’s Hospital. Therefore, when constructing the prediction model, importance weighting adjustment And 1:1 oversampling balance are needed to approximate the distribution of the target domain. (Table 1).

**Table 1:** Comparison of baseline data in training set, internal validation set and external validation set of prostate biopsy patients

variable	Kunming Children’s Hospital (*n*=481)		external validation sets Peking University Hospital (*n*=132)	*P*
	training sets (*n*=337)	Internal validation sets (*n*=144)		
Hydrocephalus				<0.001
Yes	38(11.28)	22(15.28)	51(38.64)	
No	299(88.72)	122(84.72)	81(61.36)	
Gestational age				<0.001
Term(≥37w)	277(82.20)	129(89.58)	89(67.42)	
Preterm(<37w)	60(17.80)	15(10.42)	43(32.58)	
Convulsions				<0.001
Yes	32(9.50)	13(9.03)	55(41.67)	
No	305(90.50)	131(90.97)	77(58.33)	
Anterior fontanel				<0.001
Bulge	28(8.31)	16(11.11)	27(20.45)	
Flat and soft	309(91.69)	128(88.89)	105(79.55)	
Gestational age				<0.001
Term(≥37w)	277(82.20)	129(89.58)	89(67.42)	
Preterm(<37w)	60(17.80)	15(10.42)	43(32.58)	
Amniotic Fluid				0.380
Normal	303(89.90)	124(86.10)	118(90.80)	
Meconium-stained	34(10.10)	20(13.90)	12(9.20)	
Intracranial hemorrhage				0.807
Yes	100(29.67)	46(31.94)	42(32.30)	
No	237(70.33)	98(68.06)	88(67.70)	
The lowest CSF glucose Level				<0.001
<2mmol/L	122(36.20)	63(43.75)	96(72.73)	
≥ 2mmol/L	215(63.80)	81(56.25)	36(27.27)	
Peak level of CSF protein				<0.001
>1.7 g/L	310(92.0)	132(91.70)	43(33.10)	
≤ 1.7 g/L	27(8.0)	12(8.30)	87(66.90)	

### Materiality weighting adjustment

In this study, the external validation set was statistically different from the training set on several variables. To balance this difference, a multivariate logistic regression model was constructed with the training set as the target distribution through the R language weightit package. The data included in the two hospitals were used as dependent variables, and variables with statistically significant differences at baseline were used as independent variables to calculate the propensity score weights for each sample in the external validation set.

**Fig. 2:**
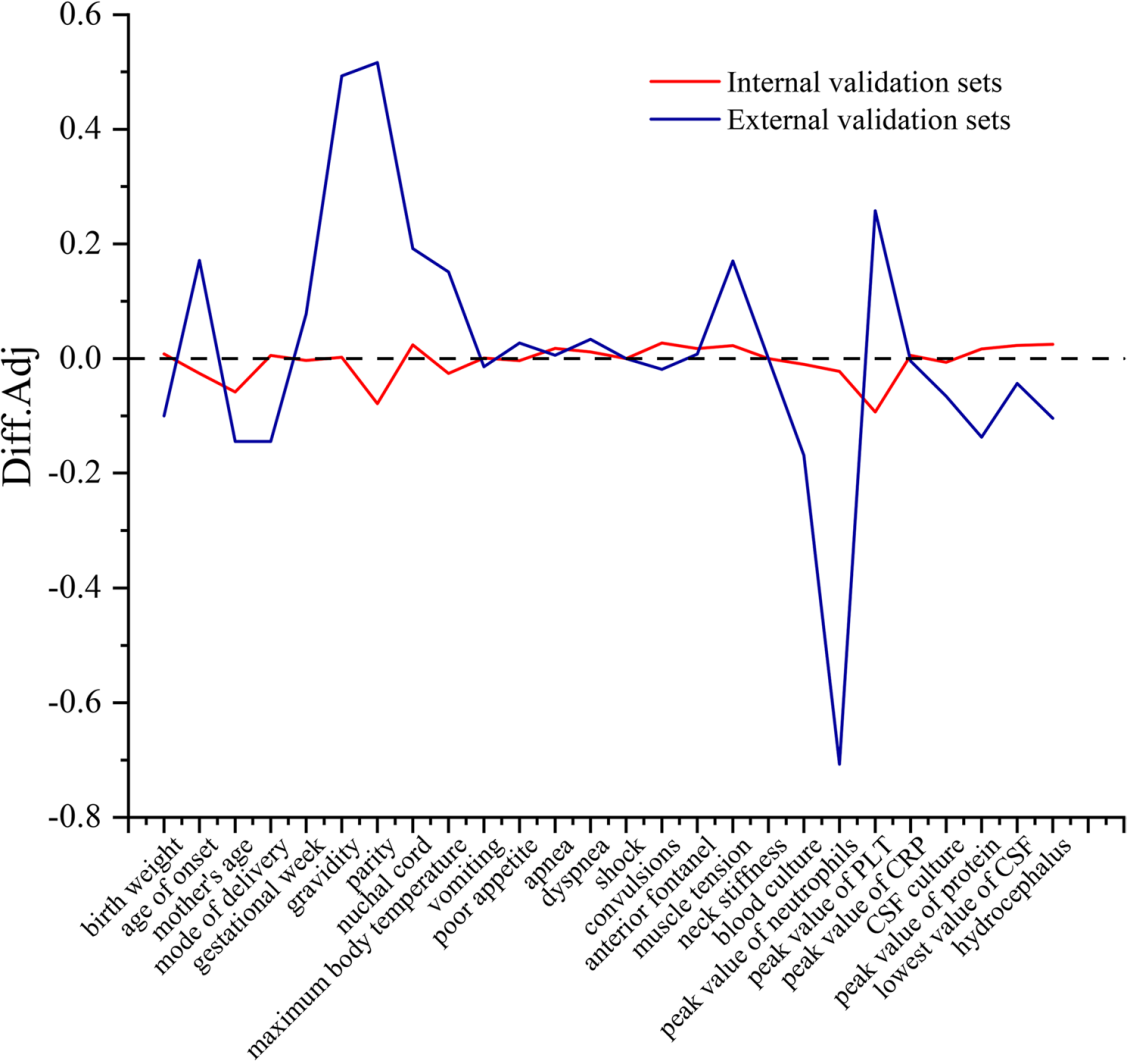
Distribution of Propensity Score Weights in Internal and External Validation Sets

The Diff Adj of most indicators in the internal validation set tends to be close to 0, indicating that they were highly matched with the baseline distribution of the training set, and the differences had been effectively balanced after weighting. Some indicators in the external validation set still had non-zero differences, reflecting the inherent difference between their original baseline characteristics and the training set, but the overall difference amplitude had been significantly reduced by weighting. The clinical heterogeneity of the multicenter data was retained, and the interference of baseline differences on the model was narrowed by weighting, which provided a data balance basis for cross-center model validation and ensured that the subsequent analysis of the applicability of the prediction model in different medical centers was more reliable.These patterns are visualised in Fig. 2.

**Fig. 3:**
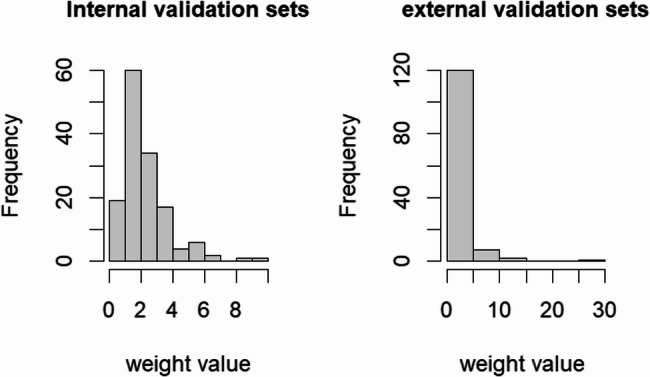
Distribution Histogram of Weight Values in Internal and External Validation Sets

The weights of the internal validation set are concentrated in the range of 0–6, And most of the samples had relatively uniform weight distributions, so they could participate in the model construction without significant adjustments. The weights of the external validation set were mainly around 0, but there were a small number of high-weight samples, and the distribution of the training set was forced to be aligned with the high weight, so as to effectively reduce the center of the bias on the interference of the model. These patterns are visualised in Fig. 3.

### LASSO regression screening variables

**Fig. 4:**
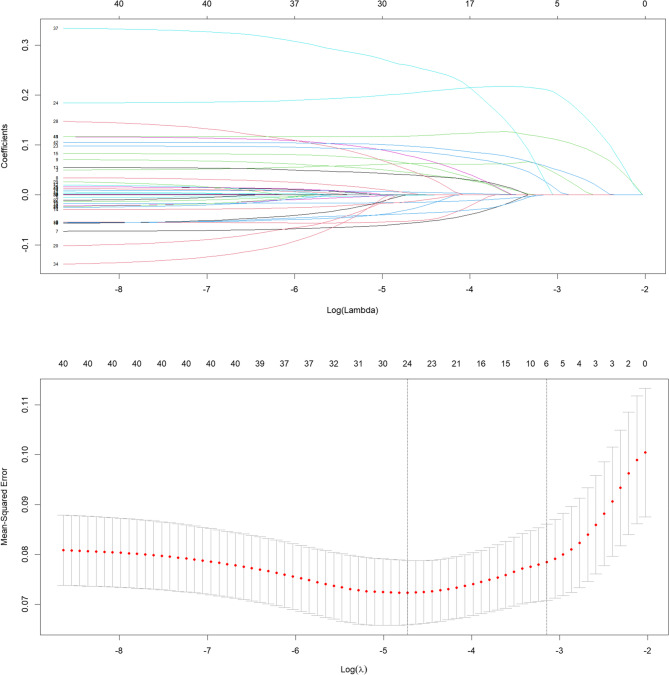
Selection of predictors of prostate biopsy using the LASSO binary logistic regression model. Note: **A** represents the coefficient curve of 32 variables, and **B** represents the process of screening the optimal λ

Based on the basic information, clinical manifestations, And laboratory results of the children in the training set, the LASSO regression in R programming language was used to screen the independent risk factors among the 32 predictive variables. The coefficients were meaningful when they were non-zero. When λ = 0.012, the model had the minimum mean value of the objective parameter. When λ = 0.068, the simplest model was obtained within one standard deviation of the minimum λ value. At this time, Logλ = − 4.450, the regression model was considered optimal. A total of 20 predictive variables could be included, including NBM type, birth weight (BW), age of onset, pregnancy complications, gestational age, birth asphyxia, umbilical cord, amniotic fluid, maximum body temperature, vomiting, convulsions, anterior fontanel, multiple drug resistance in blood culture, PLT < 100 × 10⁹/L, peak value of blood WBC, the peak value of blood N, peak value of PLT, the peak percentage of CSF multinucleated cells, the lowest value of CSF glucose, and combined intracranial hemorrhage (Fig. 4).

### Multivariate logistic regression analysis for further variable screening

In this study, the incidence of NBM complicated with hydrocephalus in Kunming Children’s Hospital was smaller than that in Peking University First Hospital. To address the interference caused by the difference between the two hospitals on the model performance, 1:1 oversampling was performed to balance the outcome events to reduce the central bias before multifactorial logistic regression continued to screen the risk factors. Multifactorial logistic regression Analysis was performed with the outcome of NBM complicated with hydrocephalus as the dependent variable And the 20 predictor variables screened by the above LASSO regression as the independent variables, which showed that CSF glucose nadir, anterior fontanel, combined intracranial hemorrhage, gestational week, pregnancy complications, convulsions, amniotic fluid, and CSF peak multinuclear cells were the independent risk factors for NBM complicated with hydrocephalus (*P* < 0.05) (Table 2).

**Table 2:** Multivariate logistic regression analysis of risk factors of NBM complicated with hydrocephalus

Variable	*P*-value	OR	95% CI	VIF
Intracranial hemorrhage	<0.001	6.922	3.470-13.809	1.221
Anterior fontanel	<0.001	8.002	3.554–18.013	1.124
Minimum CSF glucose	<0.001	0.416	0.294–0.590	1.226
Gestational age	0.009	0.870	0.785–0.966	1.330
Pregnancy complications	0.012	0.284	0.107–0.757	1.171
Convulsions	0.018	2.906	1.203–7.023	1.211
Amniotic fluid	0.026	2.417	1.109–5.265	1.121
Peak multinuclear cells	0.035	1.011	1.001–1.021	1.202
Peak WBC	0.119	0.983	0.961–1.005	1.161
Umbilical cord	0.181	0.600	0.284–1.267	1.183
Vomiting	0.223	1.952	0.666–5.721	1.134
Maximum body temperature	0.514	1.087	0.846–1.395	1.376
Thrombocytopenia	0.675	0.755	0.204–2.802	1.217
Peak PLT	0.722	0.9997	0.998–1.001	1.229
Blood multidrug resistance	0.846	1.319	0.080-21.689	1.093
Age at onset	0.892	0.997	0.960–1.036	1.483
Birth asphyxia	0.948	1.027	0.466–2.261	1.264
Peak neutrophil count	0.956	0.9995	0.981–1.018	1.243

### Predictive model construction

The data of the above meaningful variables were entered into R language, And the binary logistic regression Analysis was applied, which could result in 8 regression coefficients and P-values affecting the corresponding NBM-complicated hydrocephalus, of which the P-values of CSF multinucleated cell peak, maternal pregnancy comorbidities, and convulsions were all > 0.05 (Table 3). The final remaining 5 independent risk factors were assigned to construct a risk prediction model (Table 4), Z (risk prediction model for NBM complicated with hydrocephalus) = 1. 77 anterior fontanel-1.23 CSF glucose minimum < 2.2 mmol/L + 2.11 combined intracranial hemorrhage − 0.20 gestational weeks + 1.43 amniotic fluid + 5.66.

**Table 3:** Multivariate analysis of risk factors for risk factors of NBM complicated with hydrocephalus

Variable	Coef	S.E.	Wald Z	*P*-value
Anterior fontanel	1.7685	0.5761	3.07	0.002
Minimum CSF glucose	−1.2278	0.3266	−3.76	< 0.001
Intracranial hemorrhage	2.1126	0.5148	4.10	< 0.001
Peak multinuclear cells	0.0039	0.0089	0.44	0.663
Gestational age	−0.2015	0.0807	−2.50	0.013
Amniotic fluid	1.4329	0.6211	2.31	0.021
Pregnancy complications	−1.1659	0.8955	−1.30	0.193
Convulsions	1.0317	0.6679	1.54	0.123
Constant	4.9933	2.8330	1.76	0.780

**Table 4:** Assignment table of independent risk factors for NBM complicated with hydrocephalus

Influencing factors	Variable name	Assignment method
Minimum CSF glucose<2.2 mmol/L	X1	1 = Yes; 0 = No
Anterior fontanel bulging during course	X2	1 = Yes; 0 = No
Complicated with intracranial hemorrhage	X3	1 = Yes; 0 = No
Prematurity	X4	1 = Yes; 0 = No
Amniotic fluid contamination	X5	1 = Yes; 0 = No

**Fig. 5:**
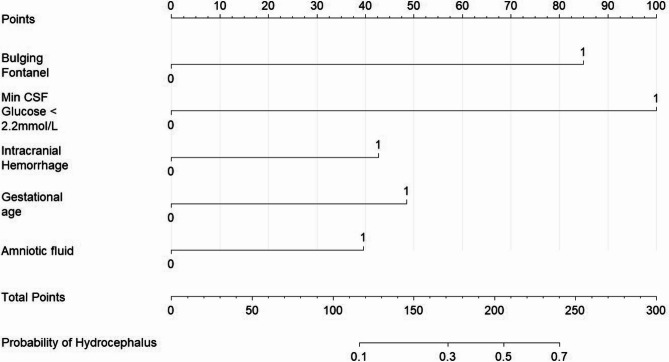
Nomogram predicting NBM complicated with hydrocephalus

A Nomogram was drawn based on the risk prediction model (Fig. 5). Neonatologists can score the Nomogram according to the corresponding indicators of the child, and calculate the estimated probability of hydrocephalus complication in children with NBM based on the projection of the total scores of the five indicators on the probability line. When a child with NBM is seen in the clinic, the relevant indicators can be quickly collected and entered to quickly determine whether the child is at high risk for NBM complicating hydrocephalus. For high-weighted indicators such as anterior fontanel, CSF glucose minimum value < 2.2mmol/L, and whether combined with intracranial hemorrhage, early intervention measures can be taken when the probability of NBM complicating hydrocephalus is found to be high. It also could be embedded in the electronic medical record system, linking multiple departments to respond according to the probability of collaborative response, optimizing the risk of judgment by iteratively monitoring the indicators dynamically, and replacing the experience by data quantification to accurately guide the intervention timing and program, so as to improve the efficiency and effectiveness of diagnosis and treatment.

### Validation of the prediction model

The data of the training set, internal validation set, and external validation set were used to conduct internal evaluation, internal verification, and external verification on this model.

#### Predictive efficacy

**Fig. 6:**
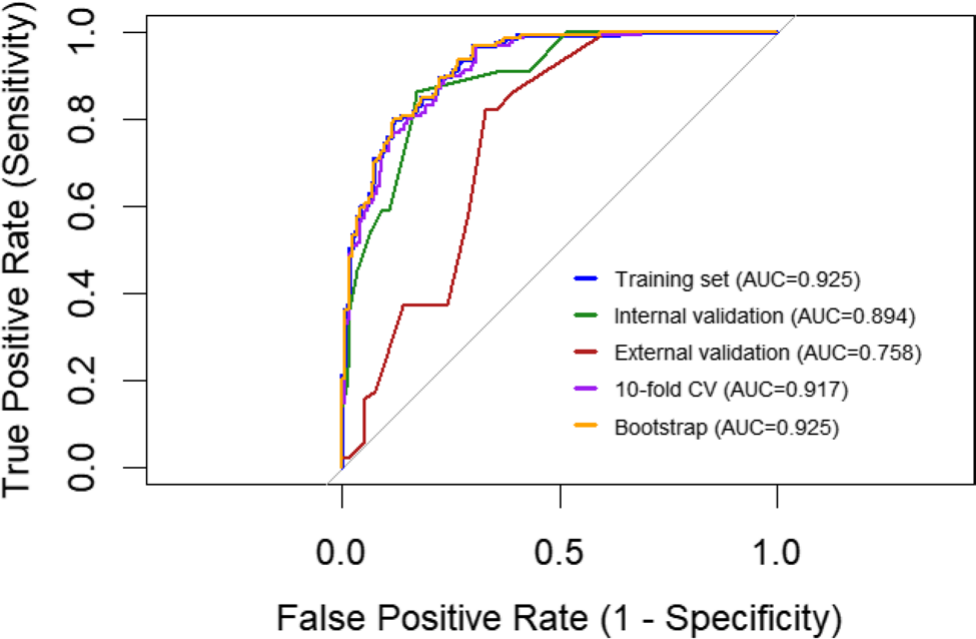
ROC curve of the nomogram for predicting NBM complicated with hydrocephalus

The ROC (Receiver Operating Characteristic) curve was used to assess discriminatory ability. The weighted results show that the area under the ROC curve (AUC) of the training set was 0.925 (95% CI = 0.899 ~ 0.951), with a sensitivity of 82.0% And a specificity of 81.0%; the AUC of the internal validation set was 0.894 (95% CI = 0.829 ~ 0.959), with a sensitivity of 84.3% And a specificity of 77.0%; And the external The AUC of the external validation set was 0.758 (95% CI = 0.677–0.839), with a sensitivity of 84.1% And a specificity of 76.8%. The 10-fold cross-validation And Bootstrap validation assisted in confirming the reliability of the model, with the former having An AUC of 0.917 And the latter having An AUC of 0.925, and the multidimensional validation demonstrated that the weighted model was effective in predicting whether or not NBM would be complicated by hydrocephalus. The training set and internal validation performed well, while the external validation was attenuated by the heterogeneity of the cohort but still had discriminatory value (Fig. 6).

#### Goodness of fit

**Fig. 7:**
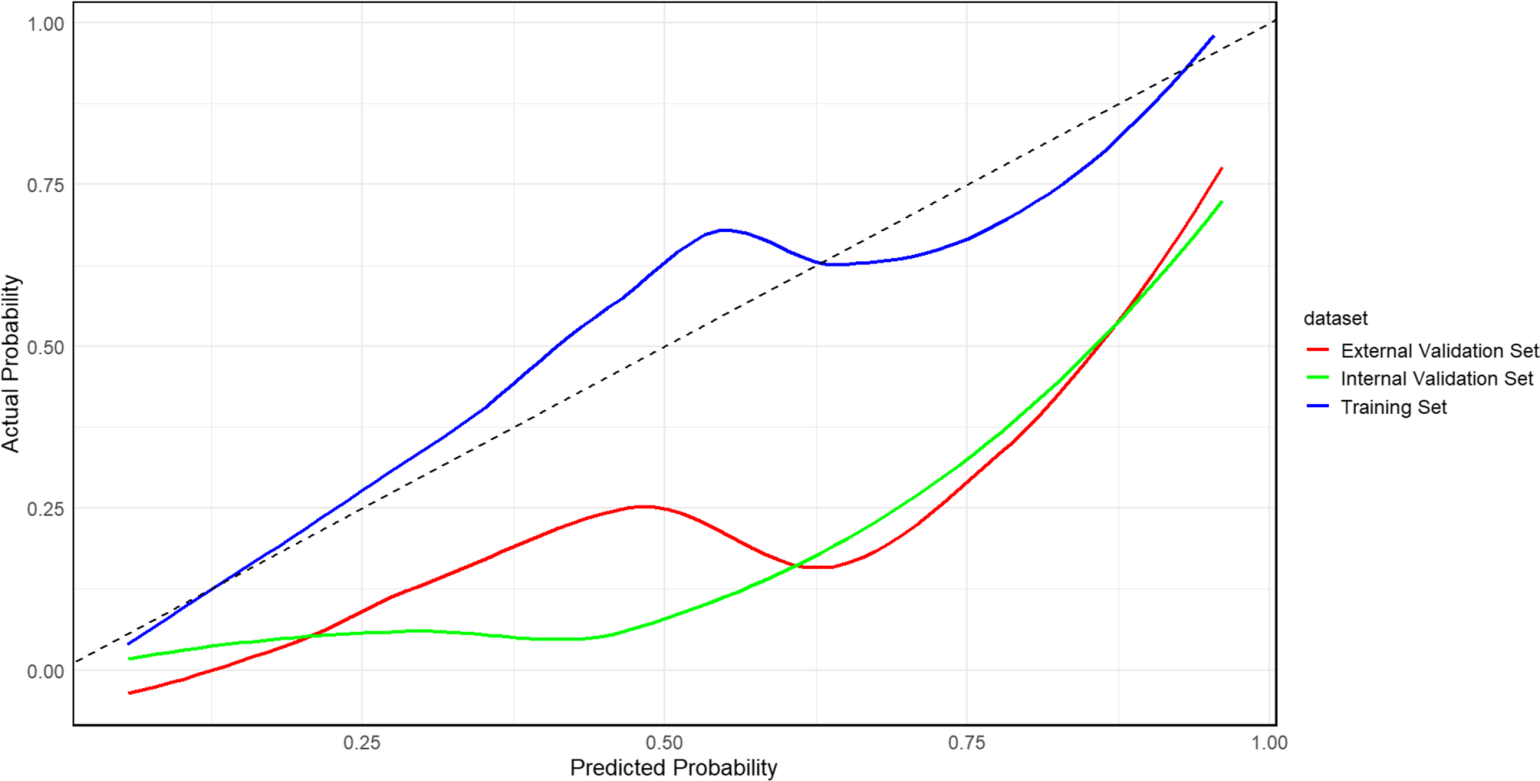
Calibration curve of the nomogram for predicting NBM complicated with hydrocephalus

The weighted results showed *P* = 0.431 for the training set, *P* = 0.224 for the internal test set, and *P* = 0.176 for the external test set, which were all > 0.05 (Fig. 7), indicating that the probability of concurrent hydrocephalus between predicted and observed NBM in the study population was generally consistent.

#### Clinical applicability

**Fig. 8:**
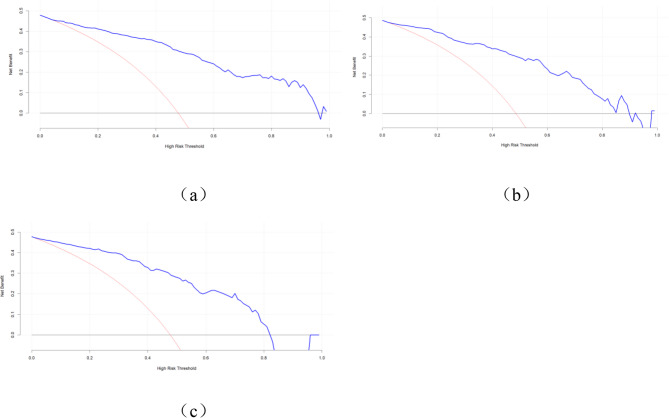
Decision curve analysis of the nomogram predicting NBM complicated with hydrocephalus. Note: In the figure, the abscissa represents the threshold probability, and the ordinate represents the net benefit value. The black line indicates the net benefit when no participants are predicted, while the red line represents the net benefit when all participants are predicted. The area between the black line and the red line in the model curve represents the clinical utility of the model. Training set (**a**), internal test set (**b**), and external test set (**c**)

The Decision Curve Analysis (DCA) was used to evaluate the clinical applicability value of the model. It can be seen from Fig. 8 that in the training set. The internal validation set, and the external validation set, the net benefit of the model was higher than that in extreme situations within a wide range of thresholds, indicating that this model has relatively high clinical applicability.

## Discussion

Neonatal bacterial meningitis (NBM) is a common infectious disease during the neonatal period. Common complications include subdural effusion, epididymitis, hydrocephalus, brain abscess, etc., but hydrocephalus is one of the more troublesome problems. The incidence rates vary in different regions [20]. CCurrently, there are not many studies related to NBM complicating hydrocephalus globally, and only a few single-center studies and studies on hydrocephalus complicating meningitis of specific pathogens [21–23]. The prognosis of NBM complicated with hydrocephalus is mostly poor. The treatment process involves multidisciplinary joint diagnosis and treatment, such as anti-infection, cerebrospinal fluid drainage, surgical intervention, etc. Even after active treatment, a few cases may still have neurological sequelae, such as cognitive dysfunction, limb motor function impairment, epileptic seizures, etc., which seriously affect the quality of life and growth and development of children. At the same time, it also brings a heavy economic burden to the families of children, making the families face great financial and psychological pressure. In this study, we retrospectively collected the basic data, clinical manifestations and laboratory results of children with NBM from Kunming Children’s Hospital and Peking University First Hospital, and analyzed the independent risk factors of NBM complicated with hydrocephalus. In this study, the incidence of NBM complicated with hydrocephalus in Kunming Children’s Hospital was 11.28%. A single-center study by Huo et al. [24]on 267 cases of bacterial meningitis in children aged < 14 years in the Department of Pediatrics, Shengjing Hospital, China Medical University, showed that the incidence of NBM complicated with hydrocephalus was 9.4%, which is similar to the present study. In this study, the incidence of NBM complicated with hydrocephalus in Peking University First Hospital was 38.64%. A single-center retrospective analysis study of risk factors for neonatal septic meningitis hydrocephalus at Fudan University Children’s Hospital by Chen et al.[13] showed that the incidence of NBM complicated with hydrocephalus was 34.01%, both of which were significantly higher than that at Kunming Children’s Hospital.

In this study, the baseline difference between Kunming Children’s Hospital and Peking University First Hospital was significant, with the latter having a higher incidence of NBM-complicated hydrocephalus, convulsions, anterior fontanel, and proportion of CSF glucose < 2 mmol/L, which was considered to be related to the degree of difficult and criticality of the hospital’s patient intake. To reduce the impact of their differences on model calibration consistency, this study addressed this issue by weighting the data with propensity scores And balancing the training set of outcome events with 1:1 oversampling. Validation showed that the weighting adjustment effectively maintained the model calibration consistency [25].

The LASSO regression Analysis in this study showed that a total of 20 predictive variables, including the type of NBM, BW, age at onset, maternal comorbidities, gestational age, birth asphyxia, umbilical cord, amniotic fluid, maximum body temperature, vomiting, convulsions, anterior fontanel, positive blood culture for multidrug-resistant bacteria, PLT < 100 × 10⁹/L, peak peripheral blood WBC, peak peripheral blood N, peak PLT, CSF peak multinucleated percentage, CSF glucose minimum, and combined intracranial hemorrhage, were risk factors for NBM complicating hydrocephalus. Lin [23]et al. similarly showed convulsions, CSF glucose levels, and birth asphyxia as risk factors in a univariate analysis of a column chart predicting hydrocephalus complicating bacterial meningitis in children aged ≥ 1 month. Liu [26] et al. in a study of risk factors for poor prognosis in NBM showed that the proportion of children with very low BW was significantly higher in the poor prognosis group compared to the good prognosis group. The occurrence of hydrocephalus is often indicative of a poor prognosis, as evidenced by peripheral blood WBC > 20 × 10^9^/L, reduced glucose content in CSF, and a significantly higher rate of positive blood cultures. The occurrence of hydrocephalus is often indicative of poor prognosis. Thus, the glucose content in CSF is closely related to the prognosis of bacterial meningitis in infants.

In the present study, CSF glucose nadir, comorbid intracranial hemorrhage, bulging fontanel, amniotic fluid status at birth and gestational week were found to be independent risk factors for concomitant hydrocephalus in NBM, which is in general agreement with the findings of several previous studies. Chen et al. [13]showed that lower CSF glucose levels were a strong predictor of hydrocephalus in children with NBM at Fudan University Children’s Hospital, and the mechanism may be related to the consumption of glucose by pathogenic bacteria that proliferate in the cerebrospinal fluid, as well as the inflammatory response that disrupts the blood-brain barrier and reduces glucose transport. A systematic review by Mao et al [27]also pointed out that CSF glucose level is not only one of the core indicators for NBM diagnosis, but the degree of its reduction is significantly correlated with poor prognosis, further supporting the clinical value of this indicator in this study. Perenc, Weller, and Deliran et al. [28–30]concluded that intracranial hemorrhage can lead to obstruction of cerebrospinal fluid circulatory pathways by inducing a local inflammatory response and promoting fibrous tissue proliferation in the subarachnoid space, which in turn increases the risk of hydrocephalus, suggesting that concomitant intracranial hemorrhage, regardless of its severity, was a predictor of poor outcome in NBM. It may be related to the stronger inflammatory waterfall response after intracranial hemorrhage due to the more immature development of brain tissue and blood-brain barrier in neonates, and the definition of intracranial hemorrhage in this study used the Papile classification of ≥ II, which is more focused on clinically significant hemorrhage. Regarding fontanel bulge some studies [31–33]illustrated that serial head circumference measurements and imaging prevented the progression of increased intracranial pressure and hydrocephalus, so regular monitoring of fontanel bulge, head circumference, and the presence of the sunset sign is essential, and cranial imaging should be repeated when necessary. Preterm infants with underdeveloped ventricular systems are more susceptible to ventricular inflammation after infection, further increasing the risk of hydrocephalus. Mao et al. [27]found that gestational age < 37 weeks was associated with poor prognosis of NBM, which was consistent with the results of this study. Amniotic fluid contamination was usually indicative of intrauterine infection or fetal distress, which may increase the probability of invasion of pathogenic bacteria into the central nervous system [7]. The present study was consistent with the findings of Liu et al. [34], who found that the incidence of amniotic fluid contamination was significantly higher in the early-onset NBM group than in the late-onset group through a single-center retrospective analysis. Studies had shown that amniotic fluid contamination was closely associated with the development of neonatal sepsis and NBM, and the severity of the infection may further promote cerebrospinal fluid circulation disorders and induce hydrocephalus [12]. A study by Mayhew et al. [35] pointed out that early-onset NBM was closely associated with intrauterine infections, which exacerbate the inflammatory response and increase the incidence of complications such as hydrocephalus, which was consistent with the results of the present study, suggesting that contaminated amniotic fluid was a potential causative factor for NBM complicated by hydrocephalus.

Based on the LASSO regression and multivariate logistic regression analysis, we developed a clinical prediction model and Nomogram including five variables of CSF glucose minimum, anterior fontanel, Prematurity, amniotic fluid contamination, and combined intracranial hemorrhage. We conducted internal verification and external verification by using the data from Peking University First Hospital. The results showed that the model had relatively high sensitivity and specificity, good fit, and high potential value in clinical practice. Clinicians can use this prediction model to conduct individualized risk assessments for children with NBM. For children at high risk of developing hydrocephalus, targeted monitoring measures can be taken earlier, such as closely observing changes in the head circumference of children, regularly performing cranial imaging examinations, regularly rechecking the routine biochemistry of CSF, and provide timely intervention treatments, such as selecting appropriate antibiotics to control infections as soon as possible and actively managing intracranial hemorrhage, to reduce the incidence of hydrocephalus and the occurrence of neurological sequelae, and improve the quality of life of children.

This study has the following limitations: ① the retrospective design led to selection bias, and the samples were obtained from two tertiary hospitals, which may not be representative of cases in primary hospitals; ② the assessment of “anterior fontanel” relies on physicians’ experience, which is subjective; ③ one fatal case was excluded due to incomplete clinical data on hydrocephalus assessment while this may slightly underestimate the risk of severe cases, the overall impact remains limited; ④ the type of pathogenic bacteria and other potential factors were not included; ⑤ differences in the diagnostic and treatment processes between the two centers may limit the extrapolation of the model. If conditions are available, further studies can be conducted to: ① carry out a multicenter prospective study, including cases in primary hospitals to verify the generalizability of the model; ② construct a time-dependent model to predict the time of onset of hydrocephalus by adding pathogenic bacteria and inflammatory factors; ③ explore the prognostic effects of interventions based on the model; and ④ replace the subjective assessment with objective indexes (e.g., ultrasound measurement of fontanel pressure) to improve the stability of the model.

In conclusion, this study constructed a prediction model for the risk of hydrocephalus complicating NBM with five predictors, including CSF glucose minimum, anterior fontanel, combined intracranial hemorrhage, gestational age, and amniotic fluid, which was internally and externally validated to show that it had a good prediction performance. It could be used to predict the risk of hydrocephalus in children with NBM and provided a reference for clinicians to follow up and reduce the neurological sequelae of NBM.

## Data Availability

The raw data supporting this study are stored in the electronic medical record systems of Kunming Children’s Hospital and Peking University Hospital. Due to ethical restrictions, these data are not publicly available. Requests for access should be directed to the corresponding author and will be subject to review by the hospital ethics committee.

## Abbreviations

NBM: Neonatal bacterial meningitis
CSF: Cerebrospinal fluid
WBC: White blood cells
PLT: Platelets
N: Neutrophil
CRP: C-reactive protein
CT: Computed tomography
MRI: Magnetic resonance imaging
ROC: Receiver operating characteristic
AUC: The area under the ROC curve
DCA: Decision Curve Analysis
